# ATF4 Is Dispensable for Spermatogenesis but Protective Against ER Stress Under Normal Conditions

**DOI:** 10.3390/biology15060466

**Published:** 2026-03-13

**Authors:** Mingxing Zhang, Zhicheng Wu, Yilan Teng, Hongwen Zhu, Peng Dai

**Affiliations:** 1 Shanghai Key Laboratory of Maternal and Fetal Medicine, Clinical and Translational Research Center of Shanghai First Maternity and Infant Hospital, Frontier Science Center for Stem Cell Research, School of Life Sciences and Technology, Tongji University, Shanghai 200092, China; zhangmx@tongji.edu.cn (M.Z.); 2031507@tongji.edu.cn (Z.W.); tengyilansh@163.com (Y.T.); 2 Precise Genome Engineering Center, School of Life Sciences, Guangzhou University, Guangzhou 510006, China

**Keywords:** *Atf4*, spermatogenesis, male fertility, integrated stress response, ER stress

## Abstract

Spermatogenesis is a metabolically intensive process that is highly sensitive to perturbations in proteostasis. The integrated stress response (ISR) and its central effector, ATF4, orchestrate adaptive responses to maintain cellular homeostasis under stress; however, the functional significance of ATF4 in mammalian spermatogenesis has not been established. To investigate this, we engineered a conditional knockout mouse model with germ cell-specific deletion of the *Atf4* gene. Results showed that *Atf4* deletion did not impair spermatogenesis or male fertility, with knockout mice exhibiting normal testicular histology and standard sperm parameters. Proteomic analysis, however, revealed that ATF4 contributes to testicular protein expression homeostasis, as its deficiency caused marked dysregulation of the testicular proteome, especially impacting SQSTM1/p62 downregulate through endoplasmic reticulum (ER) stress pathway. We conclude that ATF4’s role in regulating proteostatic balance is functionally decoupled from its necessity for the core progression of spermatogenesis. These findings define ATF4 as a potential resilience agent safeguarding testicular function under ER stress, rather than a direct regulator of male germ cell development.

## Simple Summary

Male spermatogenesis is highly sensitive to intrinsic and extrinsic environmental stress. ATF4, an essential regulator of the cellular stress reaction, is thought to contribute to maintaining reproductive health, yet its specific role in male germ cells remained unclear. In this study, we investigated whether *Atf4* plays a role in normal spermatogenesis by creating a mouse model that lacks *Atf4*, specifically in germ cells. However, these mice showed normal fertility and no obvious defects in sperm development or testicular structure. Surprisingly, proteomic analysis revealed that ATF4 contributes to maintaining the expression of endoplasmic reticulum (ER) stress-related proteins. These findings suggest that ATF4 is unlikely to directly regulate spermatogenesis but rather acts as a protective agent in mediating stress responses. These findings suggest that ATF4 acts as a potential protective agent that affects sperm survival and safeguards testicular function under ER stress.

## 1. Introduction

Male infertility represents a major global reproductive health issue, with fertility contingent on successful spermatogenesis [1]. Male infertility represents a multifactorial pathological state, with genetic determinants serving as major contributors to its etiology [2]. Mammalian spermatogenesis comprises three sequential phases: spermatogonial mitotic proliferation, spermatocyte meiosis, and spermiogenesis, which refers to the differentiation of spermatids into mature spermatozoa and involves nuclear condensation, acrosome biogenesis, and flagellum assembly. All three phases of spermatogenesis require strict spatiotemporal control to ensure normal sperm production [3,4,5].

Spermatogenesis is a highly complex and energetically demanding process characterized by rapid cell division, profound morphological transformation, and extensive protein synthesis. This inherent complexity renders it particularly vulnerable to both endogenous and exogenous stressors [6,7,8,9]. To counteract various stresses, cells have evolved a conserved integrated stress response (ISR) signaling pathway, which acts as a critical cellular surveillance mechanism and reprograms gene expression to preserve cellular homeostasis and systemic health [6,7,9]. Upon cellular stress (e.g., amino acid deprivation, proteostasis defects, oxidative insult, hyperthermia), ISR is activated, leading to kinase-mediated phosphorylation of eukaryotic initiation factor 2α (eIF2α) [7,8]. This modification attenuates global translation while selectively promoting the nuclear synthesis of activating transcription factor 4 (ATF4) is translationally induced by upstream open reading frames (uORFs) present in its mRNA transcript [7,10,11]. This paradoxical mechanism ensures the rapid and preferential synthesis of ATF4 protein during stress [12]. The CREB-family bZIP transcription factor ATF4, which binds C/EBP-ATF sites, plays a dual role in cellular fate. Not only does it regulate adaptive gene expression for processes like autophagy and ER stress relief, but its persistent activation can also trigger pro-apoptotic transcriptional programs [13,14,15,16]. Mounting studies have revealed that oxidative stress and ER stress induced by varicocele, obesity, diabetes, aging and other pathological factors exert a substantial adverse effect on spermatogenesis and male fertility by activating the ISR signaling pathway [6].

As the principal transcriptional effector of the ISR, ATF4 has emerged as a key agent in spermatogenesis of male mice [8,17,18,19]. A previous study has confirmed that global knockout of the *Atf4* gene (*Atf4*^−^/^−^) leads to abnormal constrictions in the lumen of the vas deferens, resulting in subfertility in male mice [19]. However, another study found that there is variation in the observed defects of the seminiferous tubules in *Atf4*^−^/^−^ mice: some mice exhibit almost normal spermatogenesis, while others are almost completely devoid of it [20]. Therefore, it is important to specifically knockout *Atf4* in germ cells to rule out the impact of developmental abnormalities in other tissues on individual differences in spermatogenesis.

Here, multiomics analyses encompassing single-cell sequencing demonstrated ubiquitous expression of ATF4 across mouse tissues. Germ cell-specific *Atf4* knockout mice exhibited unimpaired spermatogenesis and normal fertility. Nonetheless, *Atf4* depletion induced aberrant expression of numerous endoplasmic reticulum (ER) stress pathway-related proteins in germ cells. These observations indicate that while the loss of *Atf4* does not compromise male reproductive capacity in mice under standard laboratory settings, the perturbed protein expression profiles upon its deletion implicate a critical role for ATF4 in mediating germ cell resistance to the ISR, especially endoplasmic reticulum stress.

## 2. Materials and Methods

### 2.1. Mice

Mice on a C57BL/6J background were housed under specific pathogen-free (SPF) conditions. GemPharmatech Co., Ltd. (Nanjing, China) was the source of the (*Atf4*^flox/flox^) (Strain ID: T008417). Germ cell-specific deletion of *Atf4* was achieved by crossing *Atf4*^flox/flox^ mice with the *Stra8-GFP-Cre* transgenic line [21]. The reactions employed the following primer pairs: for the knockout allele, primers F1 (5′-TTGGCCGTATT AGGACGCGAG-3′) and R1 (5′-GCCAAATCCCAGAAGCGTCTAC-3′) yielding a 262 bp product; primers F2 (5′-TCTGAGGCGGAAAGAACCAG-3′) and R2 (5′-GTTGTACCTGTCTCCCTTAGCAGG-3′) produced a 361 bp fragment. The experimental procedures involving animals received approval from the Science and Technology Ethics Committee of Tongji University.

### 2.2. Fertility Determination in Mice

To evaluate male fertility, we used three adult wild-type (WT) and three adult *Atf4* conditional knockout (cKO) male mice. Each male was housed individually with two fertile WT adult female mice for two months. Litter sizes were then recorded from the first five pregnant females.

### 2.3. Histology, Immunofluorescence, and TUNEL Staining

The epididymis was carefully dissected from the testis using fine forceps and scissors, and separated into three distinct regions: the caput (head), corpus (body), and cauda (tail). Testes and epididymis were then fixed in Bouin’s solution for 24 h at RT or in 4% paraformaldehyde (PFA) at 4 °C overnight. Tissues were subjected to dehydration via a graded ethanol series, embedded in paraffin, and sliced into 5 μm sections with a rotary microtome. Sections fixed with Bouin’s solution were cleared in xylene (3 × 5 min) to remove paraffin, rehydrated through an ethanol gradient (100% to 70%), and stained with hematoxylin and eosin following standardized protocols.

Formalin-fixed paraffin-embedded sections underwent antigen retrieval by microwave irradiation in 10 mM sodium citrate buffer (pH 6.0) for 15 min, followed by gradual cooling to room temperature. Sections were permeabilized with 0.1% Triton X-100 in phosphate-buffered saline (PBST) for 15 min at RT, then blocked with a solution containing 10% normal donkey serum and 0.1% Triton X-100 in PBS for 60 min at room temperature. Primary antibodies diluted in blocking buffer were applied overnight at 4 °C in a humidified chamber. After three washes in PBST (5 min each), sections were incubated with species appropriate Alexa Fluor-conjugated secondary antibodies (Abbkine, A24221, A24411, Wuhan, China) and Hoechst (Beyotime Biotechnology, C1022, Shanghai, China) for 60 min at RT protected from light. Detection was performed using the following antibodies: anti-ATF4 (Beyotime, AF2560; Abways, CY5873, Shanghai, China), anti-MIWI (Proteintech, 15659-1-AP, Wuhan, China), anti-β-ACTIN (Abways, AB2001), anti-eIF2α (Proteintech, 11170-1-AP), and anti-p-eIF2α (Abclonal, AP0692, Wuhan, China), anti-DDX25 (Santa Cruz, sc-166289, Santa Cruz, CA, USA) and anti-SYCP3 (Santa Cruz, sc-136064). Apoptosis was detected using the One step TUNEL Apoptosis Detection Kit for Cells (Red Fluorescence, C1090, Beyotime Biotechnology) according to manufacturer’s protocols. All images were obtained employing an OLYMPUS CKX53SF microscope (OLYMPUS, Tokyo, Japan) and a SpinSR10 imaging system.

### 2.4. qRT-PCR

Total RNA was extracted using the Trizol (TaKaRa, T9108, Kyoto, Japan) reagent and subjected to reverse transcription for complementary DNA synthesis using the HiScript^®^ III RT SuperMix for qPCR (Vazyme, R323-01, Nanjing, China), according to the manufacturer’s instructions. mRNA abundance was determined using the UniPeak U+ One Step RT-qPCR SYBR Green Kit (Vazyme, Q226-01) on Light Cycler 96 System (Roche, Basel, Switzerland), with *Gapdh* used as the internal control for normalization.

### 2.5. Western Blot Analysis

Mouse testicular tissues were homogenized on ice in a prechilled lysis buffer (containing Tris/HCl (50 mM, pH 7.4), NaCl (150 mM), Triton X-100 (1%), a protease inhibitor cocktail, and an RNase inhibitor (both from Med Chem Express, HY-K0010, HY-K1033, Monmouth Junction, NJ, USA). Following homogenization, the samples were subjected to incubation on ice for 20 min and then centrifuged at 12,000× *g* for 10 min at 4 °C. The resulting supernatant was collected, mixed with SDS sample buffer, and boiled at 95–100 °C for 10 min. Protein samples were separated by SDS-PAGE on 10% gels and subsequently transferred to polyvinylidene difluoride (PVDF) membranes (Millipore, IPVH00010, Burlington, MA, USA). After rapid sealing, add the primary antibody, and then add the secondary antibody (Abbkine, A21020, A21010). Detection of chemiluminescent protein bands was performed on Chemiluminescent Imaging System (Tanon-5200, Shanghai, China).

### 2.6. Testis Mass Spectrometry

Following euthanasia, testes were dissected from adult mice. The harvested tissues were immediately homogenized in lysis buffer (4% SDS, 100 mM Tris-HCl, 0.1 M DTT, pH 7.6) and boiled for 10 min to achieve complete lysis and denaturation. The resulting lysates were subsequently used for both mass spectrometry analysis and Western blot.

### 2.7. Statistical Analysis

We performed all statistical analyses with GraphPad Prism software (version 10.0). Differences in testis-to-body weight ratios between WT and *Atf4* cKO mice were assessed by nonparametric tests (*t*-test) for comparisons between the two groups. Data are presented as mean ± standard deviation (SD). A *p*-value of less than 0.05 was considered statistically significant. Single-cell sequencing-based profiling of ATF4 expression across different cell types was performed using the mouse male germ cell database (https://tanglab.shinyapps.io/Mouse_Male_Germ_Cells/) (accessed on 18 February 2026) established by the Tang lab. To identify differentially expressed proteins (DEPs), the proteomic profiles of the WT and *Atf4* cKO mice (each with three biological replicates) were compared using the limma package (3.60.0) [22]. The criteria for DEPs were a fold-change greater than 1.4 and a *p*-value of less than 0.05. Furthermore, to elucidate the biological processes and pathways associated with the DEPs, Gene Ontology (GO) enrichment analysis was carried out with the DAVID tool (DAVID 2021 (Dec. 2021)) [23,24].

## 3. Results

### 3.1. ATF4 Is a Widely Expressed Protein

**Figure 1:**
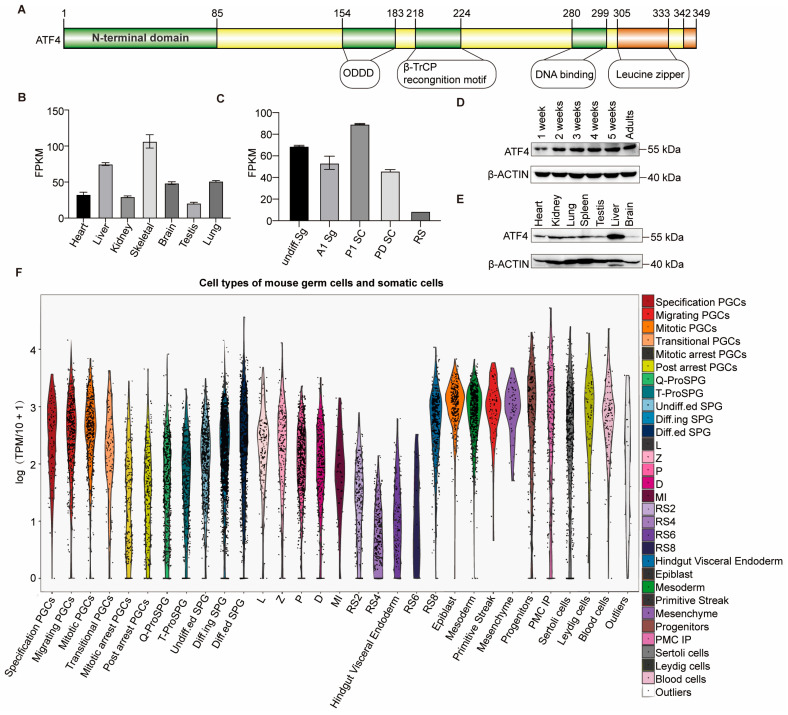
ATF4 is ubiquitously expressed in tissues under physiological conditions. (**A**) Schematic model of the ATF4 protein architecture. (**B**) Expression levels of *Atf4* (measured in FPKM, fragments per kilobase of transcript per million fragments mapped) in various tissues from adult wild type mice are presented. (**C**) The FPKM-normalized expression profiles of the *Atf4* gene across five key spermatogenic cell types are shown. The cell types analyzed include undifferentiated spermatogonia (Undiff. Sg), A1 spermatogonia (A1 Sg), preleptotene spermatocytes (PI SC), pachytene/diplotene spermatocytes (P/D SC), and round spermatids (RS). (**D**) Western blot analysis of ATF4 protein expression in mouse testes at different developmental ages (from 1 week to 5 weeks and adult mice). β-ACTIN was used as a loading control to ensure equal protein loading across samples. (**E**) Western blot analysis of ATF4 protein expression in various tissues from adult mice. A loading control used β-ACTIN. (**F**) Single-cell sequencing-based profiling of ATF4 expression across cell types.

ATF4 possessed a bipartite structure: an N-terminal transactivation domain that interacts with p300 and a C-terminal region containing the DNA-binding domain and leucine zipper for dimerization and target gene recognition (Figure 1A) [25]. Although *Atf4* expression can be activated in response to stress conditions, RNA-seq analysis reveals that *Atf4* mRNA is ubiquitously expressed across a panel of adult mouse tissues and is detectable at all stages of germ cell development under physiological conditions (Figure 1B,C). We next validated these findings at the protein level by Western blot. Protein extracts from various tissues of adult male mice and testicular tissues at different postnatal weeks were probed with an anti-ATF4 antibody, confirming ATF4 expression in all examined tissues. Moreover, ATF4 protein levels were upregulated in testicular tissue during the progression of spermatogenesis up to the spermatocyte stage (postnatal 2 weeks) (Figure 1D,E and Figures S2 and S3). Furthermore, analysis of single-cell sequencing data enabled the precise determination of ATF4 expression levels across different cell types (Figure 1F).

### 3.2. Generation of Atf4 Conditional Knockout Mice

**Figure 2:**
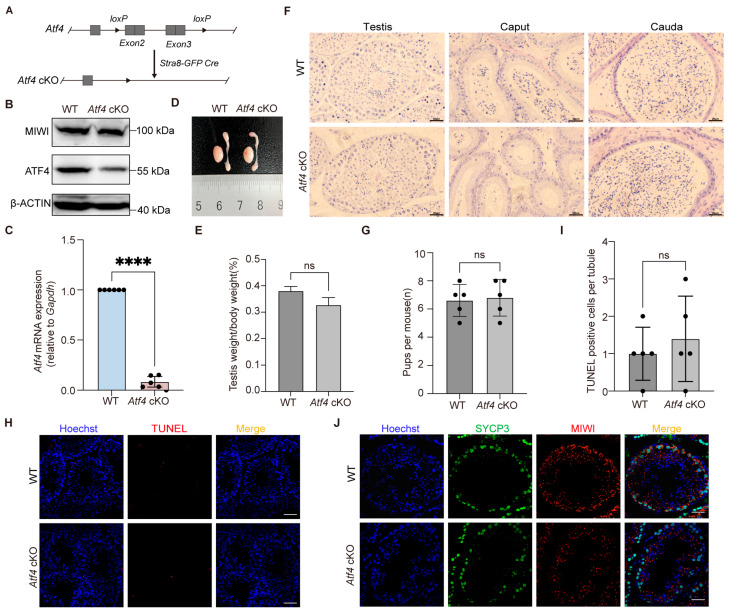
ATF4 deficiency does not impair spermatogenesis in mice. (**A**) Schematic diagram illustrating the generation of *Atf4* conditional knockout mice. LoxP sites were inserted between exons 2 and 3. Mice carrying the floxed *Atf4* allele were crossed with *Stra8-GFP-Cre* transgenic mice to achieve germ cell-specific ablation of *Atf4*. (**B**) Western blot analysis of ATF4 knockout efficiency in testicular tissues from *Atf4* cKO mice and WT littermate controls. MIWI, a marker protein for germ granules, was analyzed to evaluate the effect of *Atf4* ablation on germ granule formation and maintenance. β-ACTIN was used as the loading control to ensure equal protein loading. (**C**) qRT-PCR validation of *Atf4* transcript ablation (*Gapdh* normalization, *n* = 6, ****: *p* < 0.0001). (**D**) Gross morphology of testes and epididymides from WT and *Atf4* cKO mice. (**E**) Testis/body weight ratios showing no significant differences (unpaired *t*-test, *n* = 3 mice/group; ns: *p* > 0.05). (**F**) Hematoxylin and eosin (H&E) staining of testicular seminiferous tubules and epididymal sections. Scale bars: 20 μm. (**G**) The number of pups per mouse from WT and *Atf4* cKO mice (*n* = 5, ns: *p* > 0.05). (**H**) TUNEL assay detecting apoptotic cells in testicular sections from WT and *Atf4* cKO mice. Scale bars: 20 μm. (**I**) Quantification statistics of TUNEL positive cells per tubule (*n* = 5, ns: *p* > 0.05). (**J**) Immunofluorescence co-staining of germ cell markers in adult WT and *Atf4* cKO testis sections. Hoechst (blue), MIWI (red) and the germ cell marker SYCP3 (green), Scale bars: 10 μm.

To investigate the function of ATF4 in the reproductive system, we used CRISPR/Cas9 technology to insert loxP (flox) sequences into the intronic regions upstream of exon 2 and downstream of exon 3 on mouse chromosome 15. This targeted genomic region spans exons 2–3 and harbors all coding sequences indispensable for ATF4 function. The floxed mice were subsequently crossed with *Stra8-GFP-Cre* transgenic mice to obtain *Atf4* knockout animals specifically in male germ cells (Figure 2A). Subsequent Western blot and qRT-PCR analyses verified markedly reduced levels of *Atf4* transcript and protein in the cKO mice compared to WT controls. The residual ATF4 signal detected by Western blot in whole testis lysates is expected and derived from prespermatogonia, somatic cells (Sertoli cells, Leydig cells, etc.), and germ cells in which Cre-mediated recombination has not yet occurred (Figure 2B,C and Figure S1).

### 3.3. Normal Spermatogenesis in Atf4 cKO Mice

To assess spermatogenesis in *Atf4* cKO mice compared to WT controls, we first noted that the cKO mice were viable and exhibited no gross developmental abnormalities. We observed no significant differences in body weight or testis weight between the two genotypes (Figure 2D,E). Histological examination of testis and epididymis sections revealed that the loss of ATF4 did not discernibly affect seminiferous tubule architecture, germ cell abundance, or mature sperm production (Figure 2F). The number of progeny generated through continuous mating did not differ significantly between the *Atf4* cKO and WT mice (Figure 2G). Consistent with these findings, we detected no evidence of apoptotic signaling in the testes of *Atf4* cKO mice, indicating that germ cell development proceeded without apparent disruption under standard laboratory conditions (Figure 2H,I). Previous studies have established that the integrity of germ granules, membraneless organelles, are fundamental to spermatogenesis. These structures are thought analogous to stress granules formed via the ISR pathway. We therefore investigated the effects of *Atf4* cKO on germ granules [26,27]. Western blot analysis revealed that the level of MIWI, a germ granule marker protein, was unchanged in the testes of *Atf4* cKO mice compared with WT controls (Figure 2B). In line with this, immunofluorescence staining revealed no obvious alterations in the morphology and integrity of germ granules within the seminiferous tubules of *Atf4* cKO mice (Figure 2J). Our results indicate that *Atf4* is dispensable for spermatogenesis in mice. However, the possibility of subtle defects that were not detected in our analysis.

### 3.4. Proteomic Profiling Reveals Altered Testicular Protein Expression in Atf4 cKO Mice

**Figure 3:**
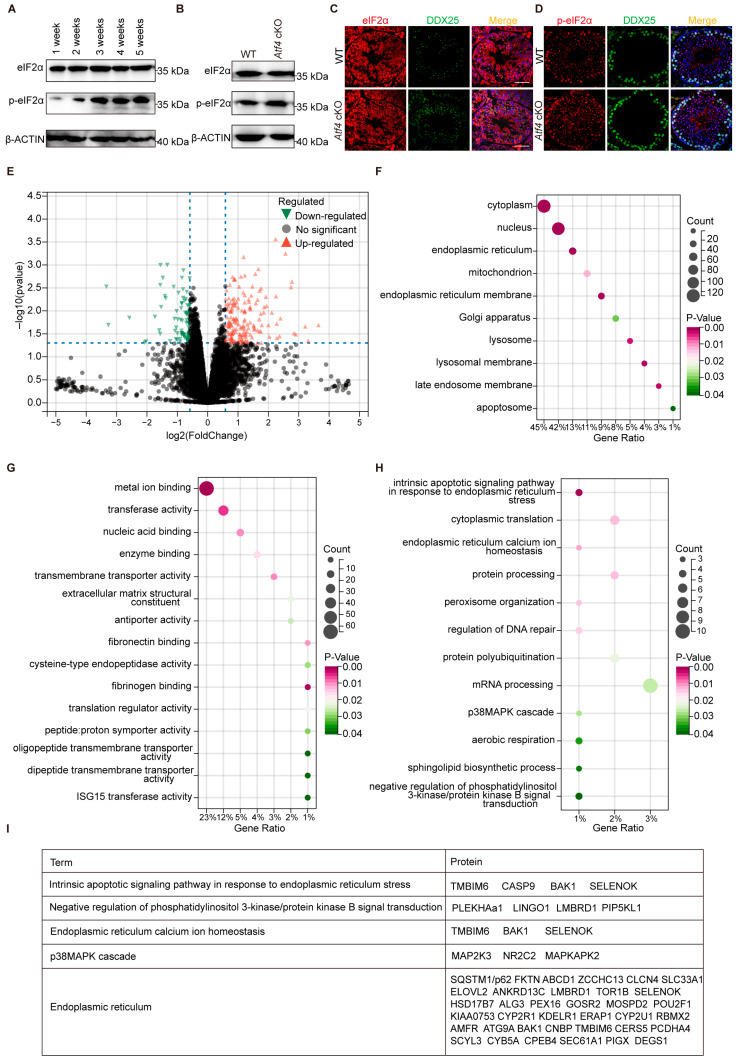
Identification of proteomic and functional changes in testes upon *Atf4* knockout. (**A**) Western blot analysis of total eIF2α and p-eIF2α protein expression in mouse testes at different developmental ages (from 1 week to 5 weeks). β-ACTIN was used as a loading control to ensure equal protein loading across samples. (**B**) Western blot analysis was performed to detect the expression levels of total eIF2α and p-eIF2α in testicular tissues from *Atf4* cKO mice and WT littermate controls. β-ACTIN was used as a loading control to ensure equal protein loading across samples. (**C**,**D**) Immunofluorescence staining of eIF2α (**C**) or p-eIF2α (**D**) in adult WT and *Atf4* cKO testis sections. Hoechst (blue), eIF2α and p-eIF2α (red) and the germ cell marker DDX25 (green), Scale bars: 10 μm. (**E**) Volcano plot identifying proteins differentially expressed in *Atf4* cKO testes compared to WT controls. Pink and green dots represent significantly up- and down-regulated proteins, respectively. Differential expression analysis was performed using the limma package. Proteins with *p*-values < 0.05 and fold changes > 1.4 were selected as differentially expressed proteins. Vertical and horizontal blue dotted lines indicate the thresholds for statistical significance (− log_10_ (*p*-value) = 1.4, equivalent to *p* < 0.05) and minimal fold-change (|log_2_ (fold change)| = 0.5), respectively. (**F**–**H**) Functional profiling of the altered proteome through GO term enrichment analysis for (**F**) cellular component, (**G**) molecular function, and (**H**) biological process. (**I**) List of differentially expressed proteins involved in ER stress-related terms in *Atf4* cKO testes compared with WT testes.

To further investigate the role of ATF4, we examined its upstream regulator eIF2α and found that total eIF2α protein levels remained unchanged, whereas p-eIF2α levels were upregulated in testicular tissue during spermatogenesis up to the spermatocyte stage (postnatal 2 weeks) (Figure 3A and Figure S2). This aligns with the expression characteristics of ATF4, demonstrating that ATF4 expression is likewise modulated by p-eIF2α throughout spermatogenesis (Figure 1D). Western blot analysis showed that while the total eIF2α level was unaltered, p-eIF2α was slightly elevated in the testes of *Atf4* cKO mice (Figure 3B and Figure S1). Consistent with this, immunofluorescence staining showed no alteration in the spatial distribution or intensity of eIF2α, and slightly increased intensity of p-eIF2α in the seminiferous tubules of *Atf4* cKO mice (Figure 3C,D). Deletion of *Atf4* affects the phosphorylation of its upstream regulator eIF2α, which we propose may represent a protective compensatory response.

To explore the impact of *Atf4* deficiency on ISR downstream pathways, proteomic analysis of *Atf4* cKO mouse testes identified extensive protein expression alterations, including 282 differentially expressed proteins relative to WT controls (Table S1). Among these, 180 proteins were significantly up-regulated while 102 were down-regulated, indicating a substantial rewiring of the testicular proteome upon *Atf4* depletion (Figure 3E). Subsequent GO analysis of the differentially expressed proteins showed significant enrichment of ER stress-related terms, including the intrinsic apoptotic signaling pathway in response to ER stress, ER calcium ion homeostasis, the p38 MAPK cascade, and negative regulation of PI3K/Akt signal transduction (Figure 3F–I). These findings strongly suggest a close association between *Atf4* deletion and ER stress pathway.

## 4. Discussion

Spermatogenesis is a complex differentiation process tightly regulated by gene expression, driven by nearly 2000 genes with testis-enriched expression [17,28]. Despite the identification of many genes essential for male fertility through knockout models, an equally substantial number prove to be dispensable. This functional dichotomy underscores the necessity of precise genetic characterization to delineate their roles in germ cell development.

Previous studies have implicated various stress conditions in impaired spermatogenesis and male infertility. The activation of stress responses by these insults often leads to reproductive deficits [6,9,18]. This is corroborated by findings in patients with oligo as the amenorrhea, where altered expression of ISR components (e.g., p-eIF2α, ATF4, CHOP) correlates with infertility, suggesting a direct link between ISR dysregulation and reproductive failure [29,30]. As arm of the ISR, ER stress induces ATF4 synthesis through eIF2α phosphorylation [31]. ATF4 then maintains cellular homeostasis through a key mechanism that involves upregulating the expression of autophagy-related, antioxidant defense, and amino acid metabolism genes to ensure cell survival. In contrast, its dysregulated activation further drives the expression of pro-apoptotic genes [13,14,15,16,32,33,34]. Functioning as a central node integrating diverse cellular stresses with adaptive transcriptional programs, ATF4 likely serves a protective role in the reproductive system under standard laboratory conditions.

However, previous studies have reported inconsistent findings regarding the effects of global *Atf4* knockout on spermatogenesis [19,20]. We hypothesize that this discrepancy may arise from differences in mouse generation strategies, housing conditions, or indirect effects of ATF4 deficiency in other tissues. We therefore utilized a germ cell-specific conditional *Atf4* knockout mouse model to directly investigate the intrinsic role of ATF4 in spermatogenesis.

We demonstrate that ATF4 is expressed not only in various tissues but also in all types of germ cells throughout spermatogenesis, coinciding with the stage of spermatogenesis that is most vulnerable to both intrinsic and extrinsic stressors. However, our results indicate that the loss of *Atf4* does not confer an overt reproductive phenotype under standard laboratory conditions, with spermatogenesis and male fertility remaining largely unaffected. Interestingly, the absence of *Atf4* led to significant alterations in the testicular proteome, specifically affecting the ER stress pathway. Previous studies have suggested that ER stress upregulates SQSTM1/p62 through the ATF4-dependent pathway [35]. Notably, our data demonstrate that knockout of ATF4 resulted in a marked decrease in SQSTM1/p62 protein expression (Table S1). The IRE1α/XBP1 and ATF6 pathways represent two additional critical branches of the ER stress response. These pathways transcriptionally upregulate proteins that facilitate proper protein folding or mediate the degradation of misfolded proteins, thereby relieving ER stress and restoring ER homeostasis [35]. Mass spectrometry analysis revealed that, among the detected proteins, loss of *Atf4* did not alter these two ER stress pathways (Table S1). Therefore, under *Atf4* cKO conditions, the changes in ER stress pathway proteins are mainly attributable to alterations in the PERK-p-eIF2α-ATF4 axis. However, we cannot exclude the possibility that the PERK-p-eIF2α-ATF4 axis acts cooperatively with the IRE1α/XBP1 and ATF6 pathways when testicular tissue is exposed to ER stress.

Definitive assessment of the functional interplay within the ISR pathway will require combinatorial gene targeting approaches to circumvent compensatory mechanisms. Taken together, our data demonstrate that *Atf4* deficiency triggers reprogramming of the testicular proteome, with the ER stress pathway being a key target of these alterations (Figure 3H). It is therefore plausible that these underlying molecular changes could compromise fertility under challenging conditions that induce ER stress, such as metabolic dysfunction or aging. Future studies employing specific stress paradigms are warranted to directly test this hypothesis. In light of the growing concerns over environmental and lifestyle stressors contributing to male infertility, this research provides important insights into how reproductive cells cope with stress, offering a foundation for future studies aimed at preserving male fertility.

## 5. Conclusions

Germ cell-specific conditional knockout of *Atf4* did not alter testicular development, the complement of germ cell types, or the progression of spermatogenesis, indicating no overt functional deficit. However, proteomic analysis of testes with germ cell-specific *Atf4* deficiency revealed significant dysregulation in the expression of ER stress-related proteins (SQSTM1/p62). These findings demonstrate that ATF4 contributes to maintain in maintaining proteostatic homeostasis is uncoupled from its requirement for the core developmental program of spermatogenesis under baseline conditions. Given the rising incidence and diversity of stress-induced male infertility, and the challenge of fully assessing testicular function, this study investigated the role of the stress-responsive transcription factor ATF4 in spermatogenesis.

## Data Availability

The data underlying this article will be shared on reasonable request to the corresponding author.

## Supplementary Materials

The following supporting information can be downloaded at: https://www.mdpi.com/article/10.3390/biology15060466/s1, Table S1: Up- and down-regulated differentially expressed proteins in testes between *Atf4* cKO and wild-type mice; File S1: Western bolt’s original data (Figure S1: Western blot analysis was performed to detect the protein expression levels in testicular tissues from Atf4 cKO mice and WT littermate controls; Figure S2: Western blot analysis of protein expression in mouse testes at different developmental ages (from 1 week to 5 weeks and adult mice); Figure S3: Western blot analysis of ATF4 protein expression in various tissues from adult mice).
