# Supplementary material for: ATF4 Is Dispensable for Spermatogenesis but Protective Against ER Stress Under Normal Conditions
Source: Biology (Basel). 2026 Mar 13;15(6):466. doi: 10.3390/biology15060466 (PMC13023884; doi:10.3390/biology15060466)
Supplement: Supplementary file 1 [file biology-15-00466-s001.zip › File S1 western bolt-supplementary version 2.pdf]

Supplementary Materials

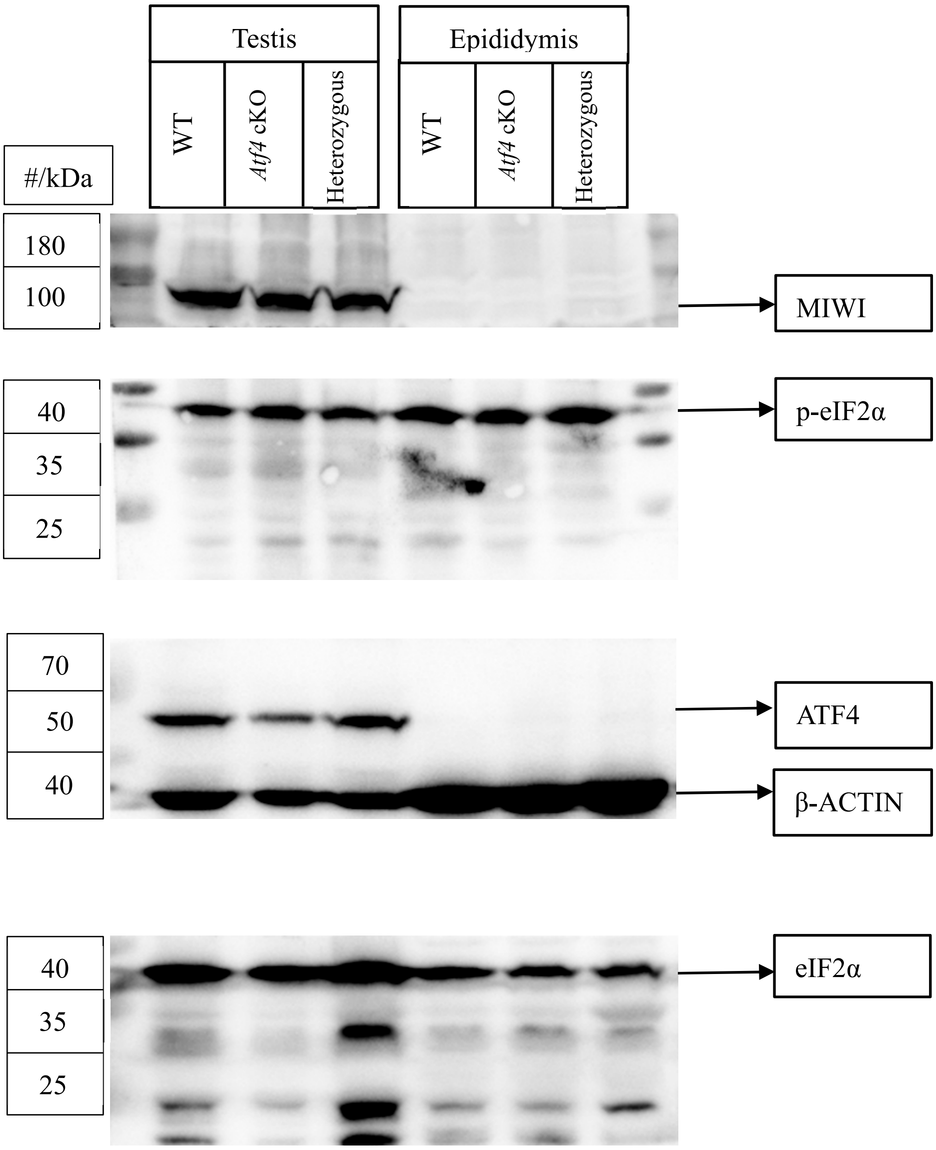

Figure S1. Western blot analysis was performed to detect the protein expression levels in testicular tissues from Atf4 cKO mice and WT littermate controls. Western blot membrane of protein detected with anti-ATF4 (~55kDa Beyotime, AF2560), anti-MIWI, (~98kDa, Proteintech, 15659-1-AP), anti-eIF2α (~36kDa, Proteintech,11170-1-AP), anti-β-ACTIN (~42kDa, Abways, AB2001), and anti-p-eIF2α (~36kDa, Abclonal, AP0692) antibody. Gel-separated proteins were transferred to polyvinylidene fluoride (0.45 μm, milipore) by electroblotting (200 mA 120 min). Membranes, incubated with a horseradish peroxidase-conjugated secondary antibody (Abbkine, A21020), were developed with Tanon™ Femto-sig ECL Western Blotting (Tanon). #Weight marker (molecular weight in kDa): Thermo Scientific™/PageRuler™ Prestained Protein Ladder, 10 to 180 kDa; catalogue number: 26616.Experiment Date：2024/06/25.

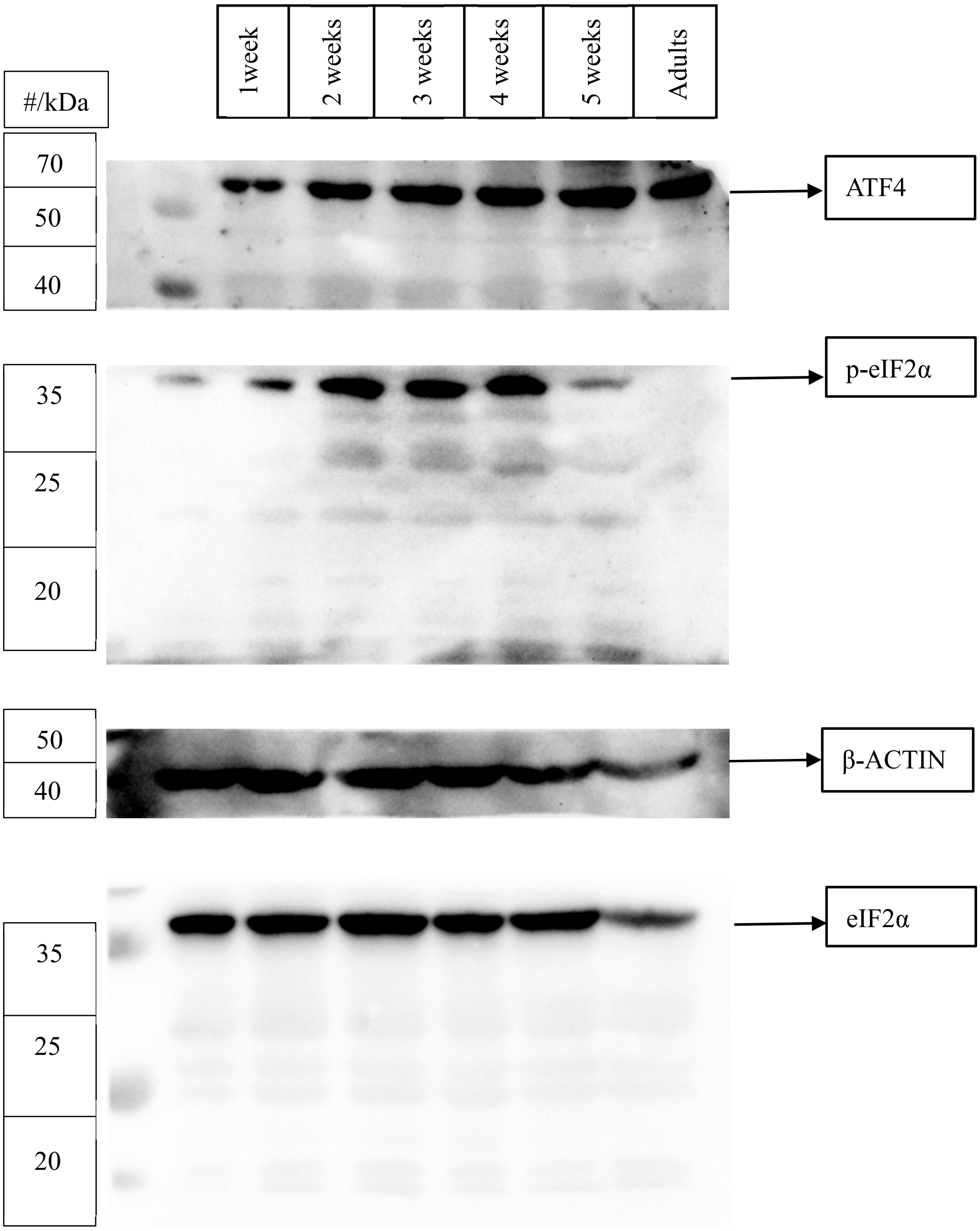

Figure S2. Western blot analysis of protein expression in mouse testes at different developmental ages (from 1 week to 5 weeks and adult mice.Western blot membrane of protein detected with anti-ATF4 (~55kDa, Beyotime, AF2560) anti-β-ACTIN (~42kDa, Abways, AB2001), anti-eIF2α (~36kDa, Proteintech,11170-1-AP) and anti-p-eIF2α (~36kDa, Abclonal, AP0692) antibody. Gel-separated proteins were transferred to polyvinylidene fluoride (0.45 μm, milipore) by semidry electroblotting (200 mA 120 min). Membranes, incubated with a horseradish peroxidase-conjugated secondary antibody (Abbkine, A21020), were developed with Tanon™ Femto-sig ECL Western Blotting (Tanon). #Weight marker (molecular weight in kDa): Thermo Scientific™/PageRuler™ Prestained Protein Ladder, 10 to 180 kDa; catalogue number: 26616. Experiment Date：2023/07/08

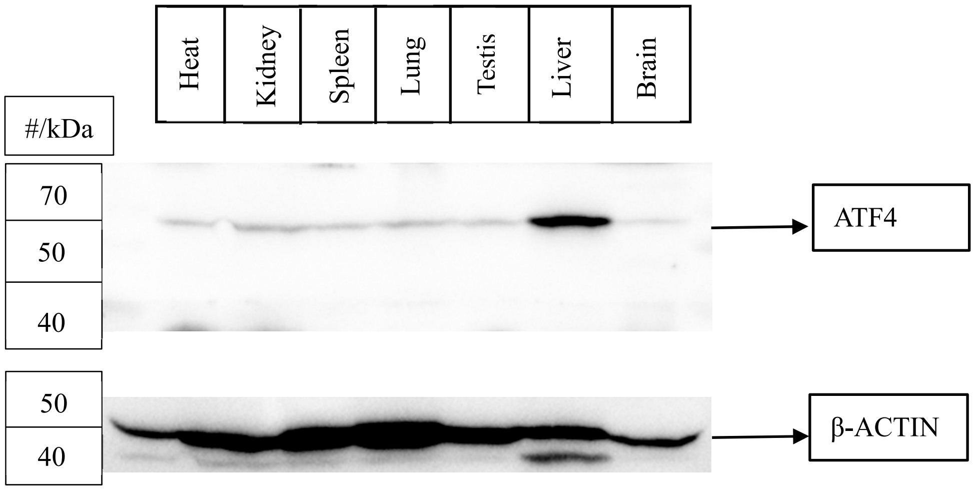

Figure S3. Western blot analysis of ATF4 protein expression in various tissues from adult mice. Western blot membrane of protein detected with anti-ATF4 (~55kDa, Beyotime, AF2560) and anti-β-ACTIN (~42kDa, Abways, AB2001). Gel-separated proteins were transferred to polyvinylidene fluoride (0.45 μm,milipore) by semidry electroblotting (200 mA 120 min). Membranes, incubated with a horseradish peroxidase-conjugated secondary antibody (Abbkine, A21020), were developed with Tanon™ Femto-sig ECL Western Blotting (Tanon). #Weight marker (molecular weight in kDa): Thermo Scientific™/PageRuler™ Prestained Protein Ladder, 10 to 180 kDa; catalogue number: 26616. Experiment Date：2024/4/27
